# Estimating the potential survival gains by eliminating socioeconomic and sex inequalities in stage at diagnosis of melanoma

**DOI:** 10.1038/bjc.2015.50

**Published:** 2015-03-03

**Authors:** M J Rutherford, L Ironmonger, N Ormiston-Smith, G A Abel, D C Greenberg, G Lyratzopoulos, P C Lambert

**Affiliations:** 1 Department of Health Sciences, University of Leicester, Leicester LE1 7RH, UK; 2 Statistical Information Team, Cancer Research UK, Angel Building, 407 St John Street, London EC1V 4AD, UK; 3 Cambridge Centre for Health Services Research, Institute of Public Health, Department of Public Health and Primary Care, University of Cambridge, Cambridge, UK; 4 National Cancer Registration Service, Public Health England, Eastern Office, Cambridge CB22 3AD, UK; 5 Health Behaviour Research Centre, Department of Epedimiology and Public Health, University College London, 1-19 Torrington Place, London WC1E 6BT, UK; 6 Department of Medical Epidemiology and Biostatistics, Karolinska Institutet, Stockholm SE-171 77, Sweden

**Keywords:** avoidable deaths, socioeconomic inequalities, sex inequalities, excess mortality models

## Abstract

**Background:**

Although inequalities in cancer survival are thought to reflect inequalities in stage at diagnosis, little evidence exists about the size of potential survival gains from eliminating inequalities in stage at diagnosis.

**Methods:**

We used data on patients diagnosed with malignant melanoma in the East of England (2006–2010) to estimate the number of deaths that could be postponed by completely eliminating socioeconomic and sex differences in stage at diagnosis after fitting a flexible parametric excess mortality model.

**Results:**

Stage was a strong predictor of survival. There were pronounced socioeconomic and sex inequalities in the proportion of patients diagnosed at stages III–IV (12 and 8% for least deprived men and women and 25 and 18% for most deprived men and women, respectively). For an annual cohort of 1025 incident cases in the East of England, eliminating sex and deprivation differences in stage at diagnosis would postpone approximately 24 deaths to beyond 5 years from diagnosis. Using appropriate weighting, the equivalent estimate for England would be around 215 deaths, representing 11% of all deaths observed within 5 years from diagnosis in this population.

**Conclusions:**

Reducing socioeconomic and sex inequalities in stage at diagnosis would result in substantial reductions in deaths within 5 years of a melanoma diagnosis.

Socioeconomic and sex differences in cancer survival outcomes are a persistent problem in the United Kingdom and Europe (Rosengren and Wilhelmsen, 2004; Rachet *et al*, 2008, 2010; Jansen *et al*, 2014). Survival inequalities are thought to, at least partially, reflect differences in stage at diagnosis, in addition to potential differences in treatment patterns and comorbidity. However, evidence quantifying the potential contribution of stage variation to survival inequalities (and therefore the size of potential gains in survival from eliminating inequalities in stage at diagnosis) is limited (Li *et al*, 2013; Rutherford *et al*, 2013b).

Melanoma survival varies across socioeconomic groups (Ellis *et al*, 2012), with worse survival among patients living in areas of greater socioeconomic deprivation. It is also known that women have higher survival from melanoma compared with men in England (Office for National Statistics, 2011) and in Europe (De Angelis *et al*, 2014). Compared with women with melanoma, men with melanoma are more likely to be diagnosed at an advanced stage, and the same is also true for patients living in more deprived compared with more affluent neighbourhoods (Lyratzopoulos *et al*, 2013a). Therefore, poorer survival outcomes for men and patients living in more deprived areas could at least partially be explained by these inequalities in stage at diagnosis of melanoma. Previous work has looked at calculating the deaths that could be postponed beyond a time point after diagnosis by completely removing inequalities or variation in survival (Abdel-Rahman *et al*, 2009; Pokhrel *et al*, 2010; Lambert *et al*, 2011; Ellis *et al*, 2012).

In this paper, we aimed to investigate the potentially avoidable mortality burden from melanoma that can be attributed to inequalities in stage at diagnosis by sex and socioeconomic group.

## Patients and methods

### Data

We analysed the time from diagnosis to death for people from the East of England with a new diagnosis of malignant melanoma (International Classification of Diseases–10 site code C43) during 2006–2010, with follow-up on mortality until 15 March 2012. As described previously (Rutherford *et al*, 2013b), data were extracted from the (former) Eastern Cancer Registration and Information Centre, a cancer registry covering a population of ∼5.7 million across the East of England region. Stage at diagnosis was assigned by medical practitioners with specialist expertise, based on clinical, imaging and pathological information according to the TNM classification (Sobin and Fleming, 1997). We combined stage groups III and IV because of small numbers. Socioeconomic status groups (one least deprived and five most deprived) were defined using national quintiles of the income domain of the Index of Multiple Deprivation 2010 score of the lower super output area of patients' residence at diagnosis (Department for Communities and Local Government, 2011). We categorised age into six groups: 30–39, 40–49, 50–59, 60–69, 70–79 and 80+ years. We tabulated the proportions in each stage group across deprivation and sex.

### Analysis

The purpose of the analysis was to estimate the number of deaths that could be postponed beyond 5 years from a diagnosis of melanoma if sex and deprivation inequalities in stage at diagnosis were eliminated. To do so, we first calculated the potential impact of eliminating gender differences alone (men attaining the stage distribution of women), then the potential impact of eliminating socioeconomic differences alone (all patients attaining the stage distribution of the most affluent quintile group), and finally the potential impact from eliminating both sex and socioeconomic inequalities combined (all patients attaining the stage distribution of the most affluent women).

We performed a complete case analysis on 5122 patients diagnosed between 2006 and 2010. We fitted a flexible parametric excess mortality model (Nelson *et al*, 2007; Lambert and Royston, 2009; Royston and Lambert, 2011) for age group, deprivation group, sex and stage at diagnosis. We allowed the effect of stage on survival to vary for men and women by including an interaction term between stage and sex in the model. We also allowed the effect of deprivation and age group to be time dependent by including interaction terms in the model between stage and a function of time since diagnosis—that is, the estimates for the excess hazard ratios were allowed to be different at differing points of follow-up time (Lambert and Royston, 2009). The complexity of the parametric forms was selected using information criteria (Rutherford *et al*, 2013a). Information on the expected mortality rates in the general population, which feeds into the model, was obtained from a life table stratified by age, sex, calendar year and socioeconomic status quintile groups specific to the East of England region (Cancer Research UK Cancer Survival Group, 2006). From this model, we calculated relative survival estimates that varied by sex, deprivation, stage and age group. We calculated age-standardised stage-specific relative survival for each deprivation and sex group.

We then followed a methodology similar to Rutherford *et al* (2013b) to calculate the avoidable deaths under three different scenarios, appropriately converting the estimates to all-cause mortality to account for competing causes of death. First, we calculated the deaths that would be postponed beyond 5 years from diagnosis by allowing men to have the same stage distribution (for each age and deprivation group) as women. Second, we calculated the same statistic by allowing patients in all deprivation groups to have the stage distribution (for each age and sex group) of the most affluent patients. We summed the estimates for men and women to show the overall impact of deprivation differences across the two genders. Finally, we calculated the deaths that would be postponed if all patients (in each age group) were to have the same stage distribution of the most affluent women.

Model-based uncertainty in the estimates was calculated using the delta method, as has previously been suggested for avoidable death estimates (Seppa *et al*, 2012). Similar to a previous analysis (Rutherford *et al*, 2013b), we used data on age and deprivation distribution for melanoma incidence in the whole of England for 2006–2010 (Extracted from CAS1409, 2014) to approximate the avoidable deaths that would be seen for England as a whole. This appropriately accounts for differences in the population structure in terms of sex, deprivation and age between England and the East of England. However, in doing this, we assume that England does not differ from the East of England in terms of other-cause mortality rates, the effect of age, stage, sex and deprivation on relative survival after a melanoma diagnosis, and the stage distributions of melanoma patients at diagnosis.

## Results

We analysed 5122 patients, after excluding 302 individuals (5.6% of the original cohort) because of missing information on stage at diagnosis. There were notable differences in stage distribution by deprivation group across all age groups for both men and women, with those in more deprived groups tending to have a higher proportion of late-stage disease (25 and 18% diagnosed at stages III–IV for men and women, respectively, among the most deprived patients and 12 and 8% for men and women, respectively, among the least deprived patients; Table 1). In addition, the proportion of patients with diagnosis at an earlier stage is higher among women across all deprivation and age group categories.

There were large differences in relative survival for patients diagnosed at stage I disease compared with those diagnosed at an advanced stage (III/IV) across all deprivation groups and either gender (Supplementary Online Material). 5-year relative survival is nearly 100% for stage I melanoma patients, meaning that patients have very little excess risk of death due to their diagnosis of melanoma. For men diagnosed at an advanced stage, the 5-year relative survival estimate is close to 40% across all deprivation groups. Therefore, eliminating inequalities in the stage distribution for melanoma patients will lead to substantial improvements in mortality up to 5 years post diagnosis.

**Figure 1:**
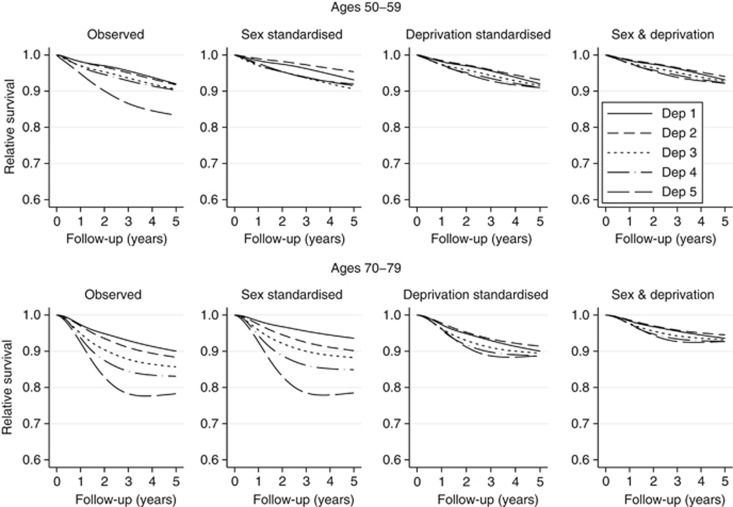
**Stage-standardised survival for two example age groups (50–59 and 70–79 years) for men with melanoma.** The left panel is stage standardised to the observed stage distribution, showing the observed survival estimates across deprivation groups. The three other panels relate to the three alternative stage standardisations. The alternative stage standardisations show the survival estimates across deprivation groups that would be achieved if the stage distribution could be improved to match that of females, the least deprived or the least deprived females, respectively.

Figure 1 shows the stage-standardised estimates of relative survival for two example age groups (50–59 and 70–79 years) for men. This shows the impact of removing inequalities in stage at diagnosis on the survival estimates, and at the same time it also illustrates inequalities in survival that would have remained if stage differences had been removed. The ‘sex-standardised' panel of Figure 1 shows the improvements for men that are seen by stage standardising to the stage distribution of women (Table 1) (within age and deprivation groups). For men aged 50–59 years, survival across all deprivation groups improves by standardising to the stage distribution observed for women, particularly for the more deprived patients. This is due to the combination of differential survival across stages for men (Supplementary Online Material) and differences in stage at diagnosis by sex in this age group, including between the most affluent men and women (Table 1). Improvements in survival are also seen for men aged 70–79 years by stage standardising to the stage distribution of women, although the effects are more modest for more deprived patients in this age category. The ‘deprivation-standardised' panel of Figure 1 shows the improvements seen by stage standardising to the stage distribution of the least deprived patients (Table 1). This has a stark impact on survival when compared with the observed relative survival estimates, particularly for the most deprived group (deprivation group 5). The pattern is consistent for the two age groups shown in Figure 1. The final panel shows the improvements for men that are seen by stage standardising to the stage distribution of the most affluent women in each age and deprivation group (Table 1). This gives the combined impact of improvement for men that would be observed if both sex and socioeconomic inequalities in stage at diagnosis were eliminated.

The results given for the ‘deprivation-standardised' panel for females (results not shown) showed a similar pattern to that for men, although the stage-specific survival was higher overall for women across all deprivation groups. It should be noted that stage standardisation by sex does not impact on estimates of potential improvements in survival between women of different deprivation groups, as this does not alter the stage distribution from that observed.

**Figure 2:**
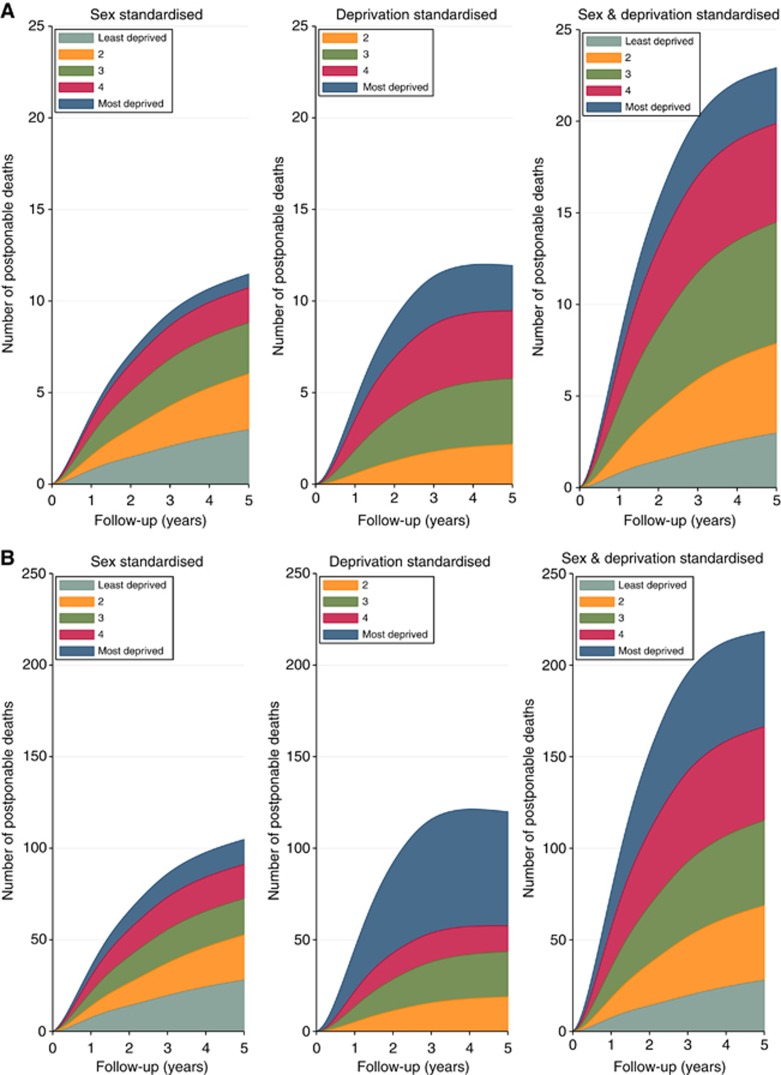
**Total number of deaths postponed beyond each time point separated by the deprivation group under the three different stage-standardisation scenarios.** (**A**) Estimates for the East of England and (**B**) England. Note that these plots are stacked and thus partition the total into constituent deprivation group contributions. The most deprived patients contribute a smaller proportion to the total in the East of England because of differences in the proportion of deprived patients in this region compared with the whole of England.

Figure 2 shows the avoidable deaths during follow-up estimated under the three explored hypothetical distributions of stage. These deaths represent the total number of deaths that would be postponed beyond each time point for a typical annual cohort size of 1024.4 (=5122/5) melanoma patients in the East of England region (Figure 2A) and 9530.4 patients (average incidence for England 2006–2010) in the whole of England (Figure 2B), under the assumptions detailed in the Patients and Methods. It should be noted that the proportion of avoidable deaths in the most deprived group is lower in the East of England compared with England as a whole. This reflects the smaller than the national average proportion of the East of England population that is socioeconomically deprived. The ‘sex-standardised' estimates are summed over all deprivation groups and show the number of deaths that are postponed if the stage distribution for men in each age and deprivation group was the same as that for women. The ‘deprivation-standardised' estimates are partitioned by deprivation group and show the total number of deaths postponed beyond each time point (summed over age and sex) if the stage distribution for all patients matched the stage distribution of affluent patients of the same sex and age group. The final panel (‘sex- and deprivation-standardised') shows the total number of deaths postponed beyond each time point under the assumption that all patients have the stage distribution of the most affluent women. These estimates are summed for men and women and across the age group.

Table 2 reports the estimates shown in Figure 2 at three time points (1, 3 and 5 years post diagnosis). The final two columns in Table 2 report the total estimates for the East of England and England as a whole. For the typical annual cohort size in England (9530.4), around 2060 deaths are estimated to occur at 5 years after diagnosis. Of those deaths, around 105, 120 and 218 deaths could have been postponed to beyond 5 years by removing stage inequalities in ‘sex only', ‘deprivation only' and ‘sex and deprivation combined', respectively. The 218 deaths that could be achieved by removing inequalities in stage at diagnosis in both sex and deprivation represent around 10.6% of the all-cause deaths that would have occurred before 5 years. Estimates for the East of England broken down by the contribution from each deprivation group are also reported with 95% confidence intervals in Table 2.

## Discussion

We have shown that eliminating differences in stage distribution for both sex and deprivation group can have a notable impact on patient survival from melanoma. We estimate that, for the typical annual cohort size of melanoma patients in England during the study period (9530), around 218 deaths (24 in the East of England) could have been postponed beyond 5 years from diagnosis if sex and deprivation differences in stage at diagnosis of melanoma were to be eliminated. This represents 10.6% of all-cause deaths among patients with melanoma predicted to occur within 5 years of diagnosis.

In this paper, we have examined the impact of improving the stage distribution of all patients to match that of population subgroups with a better overall stage mix. Women have better survival than men with melanoma even after adjusting for stage at diagnosis (Joosse *et al*, 2011). Under our approach, we calculated estimates for men using their own stage-specific survival, while matching only the overall stage distribution to that of women (using the same approach when calculating the deprivation estimates). Therefore, we provide a more realistic target for the estimate of postponed mortality than if we, for instance, assumed that the survival for men was to match that of women.

The strengths of our study include the use of highly complete and high-quality population-based information on stage at diagnosis and the use of a flexible parametric model, allowing for the smooth estimation of excess mortality throughout the follow-up period while appropriately accounting for the effects of deprivation and age (Rutherford *et al*, 2013a).

The methodology that we have used in this paper can help support monitoring of the impact of population-based interventions for earlier stage detection of melanoma (Be Clear On Cancer—Finding skin cancer early means its easier to treat, 2014). We have also appropriately accounted for competing mortality, meaning that all deaths estimated in our paper are actually postponed beyond a given time point of reporting (an alternative term used for this measure is avoidable deaths)—the entire cohort will eventually diminish to 0 if follow-up is extended long enough. National stage information in England is becoming increasingly complete, but there is currently insufficient follow-up of contemporary cohorts with highly complete information on stage at diagnosis to perform this analysis at a national level. Consequently, we have used regional data to give predicted figures for England as a whole using a weighting approach based on the relative size of the population subgroups. This approach assumes that patients with melanoma in England do not differ from those in the East of England in terms of expected survival, the effect of the covariates on relative survival and the distribution of stage at diagnosis. Overall, these assumptions appear to be fairly reasonable, particularly given the modest variation in short-term relative survival for melanoma patients between the English regions (Rachet *et al*, 2009). However, the extrapolated estimates for England should be interpreted with caution and with consideration to the stated assumptions. Uncertainty in the England estimates is further compounded by the relatively small numbers of melanoma patients with late-stage disease in the East of England, particularly for the most deprived patients, as the East of England has relatively few patients in this group compared with the national average. This meant that we were forced to combine stages III and IV, but this is unlikely to have a large impact, provided the assumption on the similarity of stage distributions between patients with melanoma in the East of England and England holds.

Our findings relate to a recent historical cohort of melanoma patients (2006–2010). Since 2011, a range of systemic treatments found to be efficacious in clinical trials are gradually being introduced into routine clinical practice for the management of advanced-stage melanoma (Corrie *et al*, 2014). It is currently too early to know whether (and by how much) new therapeutic approaches will translate into sustained improvements in longer-term survival on a population basis. However, the treatment improvements for advanced-stage disease will not eradicate the inequalities in stage at diagnosis seen in our sample. Therefore, there is still a strong case for efforts to eliminate inequalities in stage at diagnosis, particularly given the patient inconvenience, anxiety and the risks of serious side effects associated with the management of advanced disease and the appreciable healthcare costs involved (Corrie *et al*, 2014).

Temporal trends in the incidence of melanoma in the United Kingdom and Europe indicate increases in both early-stage and late-stage illness (Downing *et al*, 2006; Hollestein *et al*, 2012). This means that the estimates we have given for our study period are likely to underestimate the current potential gains that could be made. Studies have highlighted the potential for overdiagnosis of early-stage lesions that would have not otherwise been associated with morbidity/mortality during the patients' life (Weyers, 2012). There could be potential differences in the proportion of overdiagnosed cases by deprivation group and sex. This would affect the proportion of early-stage disease diagnosed in particular subgroups and result in diluting the proportions diagnosed for late-stage disease for some population groups, potentially having an impact on our estimates. Further research into variation in the overdiagnosis of melanoma by population groups is required.

The size of reductions in deaths from cancer that can result from eliminating inequalities in stage at diagnosis will reflect cancer incidence, cancer survival and the size of stage inequalities. In the case of melanoma, the majority of patients are diagnosed at an early stage (see Table 1) where the prognosis is good (see Supplementary Online Material). These factors, combined with the incidence of melanoma in England, result in the estimate of 218 deaths being postponed beyond 5 years. Although this population-wide estimated benefit might be considered modest, it should be noted that it represents around 11% of all deaths that would have occurred within 5 years among patients with melanoma, reflecting the relatively large differences in stage at diagnosis by sex and deprivation group.

Inequalities in stage at diagnosis of melanoma could result from tumour-type differences. We have examined differences in tumour type in our study population using three broad morphological types (nodular, superficial spreading and other types) and found a similar distribution of tumour type by the deprivation group for either gender. However, there are some differences in tumour-type distribution between men and women, with women having a higher proportion of superficial spreading tumour type (58 *vs* 51% for men). Furthermore, those with a superficial spreading tumour type are more likely to be diagnosed at an early stage compared with other tumour types. Therefore, these tumour-type differences by gender may limit the potential for men to attain the stage distribution of women with melanoma. Our methods also assume that anatomical site differences between genders (which are known to exist (National Cancer Intelligence Network, 2012)) are largely unrelated to survival.

In order to consider the policy implications of the findings, it is important to explore the probable principal cause of the observed differences in stage at diagnosis of melanoma. Inequalities in stage at diagnosis may reflect inequalities in diagnostic intervals post presentation between men and women and between patients with different deprivation groups. However, this is unlikely, given that diagnostic suspicion by doctors is aroused promptly in the great majority of cases with melanoma—one of the ‘easiest-to-suspect' cancers post presentation to a general practitioner (median interval from presentation to referral for patients subsequently diagnosed with melanoma of 0 days (Lyratzopoulos *et al*, 2013b). Further, there is no evidence for socioeconomic inequalities in the promptness of specialist referral after presentation to a general practitioner (Lyratzopoulos *et al*, 2012). Consequently, the observed inequalities by sex and deprivation group in stage at diagnosis of melanoma are likely to result from inequalities in the speed of help-seeking. Recorded intervals from symptom onset to presentation to a general practitioner among melanoma patients tend to be longer than those observed for patients with most other cancers (Baughan *et al*, 2009; Keeble *et al*, 2014), perhaps because of slow onset of symptoms and potential misattribution of skin changes to other causes (Walter *et al*, 2014). Further, the risk of delayed help-seeking is likely to vary for different patient groups. Psychosocial determinants of prompt presentation for potential cancer symptoms are known to exhibit a strong sociodemographic pattern, whereby, for example, men and lower socioeconomic status individuals have both lower knowledge of cancer symptoms and risk factors, and higher attitudinal or psychological barriers to prompt presentation (Macleod *et al*, 2009; Robb *et al*, 2009; Beeken *et al*, 2011; Quaife *et al*, 2014).

In conclusion, we have shown that substantial reductions in the number of early deaths from melanoma could be made if socioeconomic and sex differences in stage at diagnosis could be removed. The findings demonstrate the need for continuing development and evaluation of interventions designed to decrease intervals to presentation among patients subsequently diagnosed with melanoma. Such interventions, such as public awareness campaigns to encourage people who notice any unusual or persistent changes to their skin to visit their general practitioner (Be Clear On Cancer—Finding skin cancer early means its easier to treat, 2014), should aim to particularly encompass men, and patients of lower socioeconomic groups, as the groups at higher risk of presentation at an advanced stage.

## Figures and Tables

**Table 1:** Stage distribution across deprivation groups, separated by sex

	**Males**				**Females**			
	**Stage I**	**Stage II**	**Stage III/IV**	**Total**	**Stage I**	**Stage II**	**Stage III/IV**	**Total**
**Ages 30–39 (years)**								
Affluent	30 (75.0)	5 (12.5)	5 (12.5)	40 (100)	54 (84.4)	6 (9.4)	4 (6.3)	64 (100)
2	30 (75.0)	6 (15.0)	4 (10.0)	40 (100)	53 (82.8)	11 (17.2)	0 (0.0)	64 (100)
3	39 (95.1)	2 (4.9)	0 (0.0)	41 (100)	53 (86.9)	7 (11.5)	1 (1.6)	61 (100)
4	19 (65.5)	6 (20.7)	4 (13.8)	29 (100)	33 (75.0)	10 (22.7)	1 (2.3)	44 (100)
Deprived	7 (63.6)	3 (27.3)	1 (9.1)	11 (100)	16 (80.0)	3 (15.0)	1 (5.0)	20 (100)
**Ages 40–49 (years)**								
Affluent	62 (79.5)	12 (15.4)	4 (5.1)	78 (100)	119 (86.2)	18 (13.0)	1 (0.7)	138 (100)
2	59 (64.8)	26 (28.6)	6 (6.6)	91 (100)	103 (83.1)	13 (10.5)	8 (6.5)	124 (100)
3	59 (76.6)	9 (11.7)	9 (11.7)	77 (100)	90 (78.9)	18 (15.8)	6 (5.3)	114 (100)
4	32 (65.3)	12 (24.5)	5 (10.2)	49 (100)	47 (78.3)	9 (15.0)	4 (6.7)	60 (100)
Deprived	11 (73.3)	2 (13.3)	2 (13.3)	15 (100)	15 (78.9)	2 (10.5)	2 (10.5)	19 (100)
**Ages 50–59 (years)**								
Affluent	99 (72.8)	27 (19.9)	10 (7.4)	136 (100)	90 (76.3)	21 (17.8)	7 (5.9)	118 (100)
2	89 (70.1)	25 (19.7)	13 (10.2)	127 (100)	117 (79.6)	25 (17.0)	5 (3.4)	147 (100)
3	82 (71.9)	21 (18.4)	11 (9.6)	114 (100)	86 (71.1)	24 (19.8)	11 (9.1)	121 (100)
4	44 (74.6)	9 (15.3)	6 (10.2)	59 (100)	50 (75.8)	11 (16.7)	5 (7.6)	66 (100)
Deprived	12 (57.1)	5 (23.8)	4 (19.0)	21 (100)	14 (77.8)	4 (22.2)	0 (0.0)	18 (100)
**Ages 60–69 (years)**								
Affluent	135 (64.6)	46 (22.0)	28 (13.4)	209 (100)	104 (76.5)	18 (13.2)	14 (10.3)	136 (100)
2	135 (65.5)	46 (22.3)	25 (12.1)	206 (100)	126 (71.2)	39 (22.0)	12 (6.8)	177 (100)
3	113 (63.5)	33 (18.5)	32 (18.0)	178 (100)	101 (74.8)	20 (14.8)	14 (10.4)	135 (100)
4	45 (59.2)	22 (28.9)	9 (11.8)	76 (100)	55 (75.3)	10 (13.7)	8 (11.0)	73 (100)
Deprived	13 (50.0)	8 (30.8)	5 (19.2)	26 (100)	15 (55.6)	8 (29.6)	4 (14.8)	27 (100)
**Ages 70–79 (years)**								
Affluent	86 (62.8)	32 (23.4)	19 (13.9)	137 (100)	76 (69.7)	27 (24.8)	6 (5.5)	109 (100)
2	84 (50.6)	49 (29.5)	33 (19.9)	166 (100)	74 (59.2)	30 (24.0)	21 (16.8)	125 (100)
3	88 (50.9)	50 (28.9)	35 (20.2)	173 (100)	83 (57.2)	40 (27.6)	22 (15.2)	145 (100)
4	46 (46.5)	30 (30.3)	23 (23.2)	99 (100)	38 (53.5)	18 (25.4)	15 (21.1)	71 (100)
Deprived	12 (41.4)	7 (24.1)	10 (34.5)	29 (100)	9 (34.6)	9 (34.6)	8 (30.8)	26 (100)
**Ages 80+ (years)**								
Affluent	33 (45.8)	23 (31.9)	16 (22.2)	72 (100)	34 (47.2)	21 (29.2)	17 (23.6)	72 (100)
2	35 (45.5)	20 (26.0)	22 (28.6)	77 (100)	34 (41.5)	26 (31.7)	22 (26.8)	82 (100)
3	47 (43.5)	31 (28.7)	30 (27.8)	108 (100)	44 (44.0)	34 (34.0)	22 (22.0)	100 (100)
4	18 (29.5)	19 (31.1)	24 (39.3)	61 (100)	25 (34.2)	28 (38.4)	20 (27.4)	73 (100)
Deprived	6 (30.0)	5 (25.0)	9 (45.0)	20 (100)	9 (34.6)	8 (30.8)	9 (34.6)	26 (100)

The figures given are the number (and percentage) within each stage group.

**Table 2:** Estimates of the number of postponed deaths beyond three time points for the typical annual cohort size for the three approaches of stage standardisation for the East of England, separately by the contribution for each deprivation group

	**Number of avoidable deaths by the deprivation group (95% CI)**					**Total avoidable deaths**	
**Follow-up**	**Least deprived (1)**	**Deprivation group 2**	**Deprivation group 3**	**Deprivation group 4**	**Most deprived (5)**	**East of England**	**England**
**Sex standardisation only**							
1 year	0.79 (0.47, 1.11)	0.80 (0.48, 1.74)	1.15 (0.80, 1.50)	0.90 (0.61, 1.19)	0.31 (0.15, 0.47)	3.96	35.28
3 years	2.09 (1.58, 2.59)	2.26 (1.74, 2.78)	2.67 (2.18, 3.15)	1.91 (1.53, 2.29)	0.73 (0.53, 0.92)	9.65	86.12
5 years	3.00 (2.37, 3.65)	3.12 (2.45, 3.79)	2.91 (2.40, 3.43)	2.02 (1.61, 2.42)	0.76 (0.52, 1.01)	11.83	104.83
**Deprivation standardisation only**							
1 year	—	0.64 (0.36, 0.93)	1.33 (0.84, 1.82)	1.73 (1.17, 2.28)	1.01 (0.51, 1.51)	4.71	45.17
3 years	—	1.92 (1.42, 2.43)	3.39 (2.70, 4.09)	3.84 (3.09, 4.59)	2.66 (1.96, 3.37)	11.82	115.87
5 years	—	2.35 (1.72, 2.99)	3.81 (3.03, 4.59)	3.88 (3.09, 4.68)	2.54 (1.67, 3.40)	12.59	119.93
**Sex and deprivation standardisation combined**							
1 year	0.79 (0.47, 1.11)	1.35 (0.81, 1.90)	2.43 (1.69, 3.19)	2.39 (1.62, 3.17)	1.21 (0.61, 1.81)	8.19	76.40
3 years	2.09 (1.58, 2.59)	4.01 (3.09, 4.94)	5.98 (4.90, 7.07)	5.41 (4.35, 6.47)	3.20 (2.35, 4.06)	20.70	195.73
5 years	3.00 (2.37, 3.65)	5.12 (3.96, 6.29)	6.85 (5.62, 8.09)	5.60 (4.46, 6.74)	3.08 (2.02, 4.14)	23.67	218.42

Abbreviation: CI=confidence interval.

Total estimates for England at each of the time points are also given.
