# Supplementary figures and images for: Estimating the potential survival gains by eliminating socioeconomic and sex inequalities in stage at diagnosis of melanoma
Source: Br J Cancer. 2015 Mar 3;112(Suppl 1):S116–23. doi: 10.1038/bjc.2015.50 (PMC4385984; doi:10.1038/bjc.2015.50)

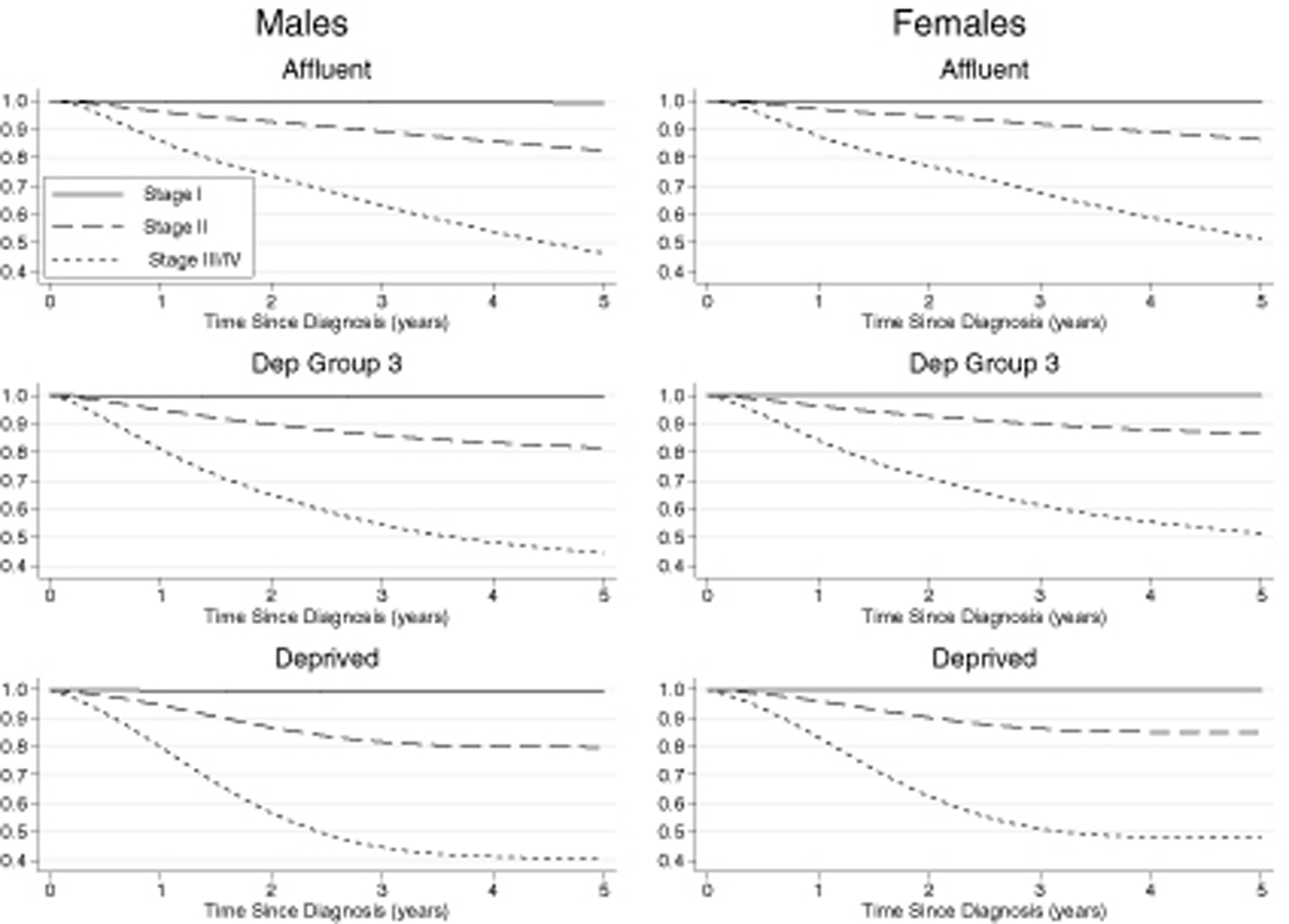

Supplement: Supplementary Figure 1 [file bjc201550x1.tif]
