# Supplementary material for: Estimating the potential survival gains by eliminating socioeconomic and sex inequalities in stage at diagnosis of melanoma
Source: Br J Cancer. 2015 Mar 3;112(Suppl 1):S116–23. doi: 10.1038/bjc.2015.50 (PMC4385984; doi:10.1038/bjc.2015.50)
Supplement: Supplementary Figure Legend [file bjc201550x2.doc]

Supplementary Figure: Internally age-standardised stage-specific survival for three deprivation groups, separately for males and females.
